# Plant growth-promoting rhizobacteria and root system functioning

**DOI:** 10.3389/fpls.2013.00356

**Published:** 2013-09-17

**Authors:** Jordan Vacheron, Guilhem Desbrosses, Marie-Lara Bouffaud, Bruno Touraine, Yvan Moënne-Loccoz, Daniel Muller, Laurent Legendre, Florence Wisniewski-Dyé, Claire Prigent-Combaret

**Affiliations:** ^1^Université de LyonLyon, France; ^2^Université Claude Bernard Lyon 1Villeurbanne, France; ^3^Centre National de la Recherche Scientifique, UMR 5557, Ecologie Microbienne, Université Lyon 1Villeurbanne, France; ^4^Laboratoire des Symbioses Tropicales et Méditerranéennes, UMR 113, Université Montpellier 2/Institut de Recherche Pour le Développement/Centre de Coopération Internationale en Recherche Agronomique pour le Développement/SupAgro/Institut National de la Recherche AgronomiqueMontpellier, France; ^5^Institut National de la Recherche Agronomique, UMR 1347, Agroécologie, Interactions Plantes-MicroorganismesDijon, France

**Keywords:** plant-PGPR cooperation, rhizo-microbiome, rhizosphere, phytohormone, plant nutrition, ISR, functional group

## Abstract

****The rhizosphere supports the development and activity of a huge and diversified microbial community, including microorganisms capable to promote plant growth. Among the latter, plant growth-promoting rhizobacteria (PGPR) colonize roots of monocots and dicots, and enhance plant growth by direct and indirect mechanisms. Modification of root system architecture by PGPR implicates the production of phytohormones and other signals that lead, mostly, to enhanced lateral root branching and development of root hairs. PGPR also modify root functioning, improve plant nutrition and influence the physiology of the whole plant. Recent results provided first clues as to how PGPR signals could trigger these plant responses. Whether local and/or systemic, the plant molecular pathways involved remain often unknown. From an ecological point of view, it emerged that PGPR form coherent functional groups, whose rhizosphere ecology is influenced by a myriad of abiotic and biotic factors in natural and agricultural soils, and these factors can in turn modulate PGPR effects on roots. In this paper, we address novel knowledge and gaps on PGPR modes of action and signals, and highlight recent progress on the links between plant morphological and physiological effects induced by PGPR. We also show the importance of taking into account the size, diversity, and gene expression patterns of PGPR assemblages in the rhizosphere to better understand their impact on plant growth and functioning. Integrating mechanistic and ecological knowledge on PGPR populations in soil will be a prerequisite to develop novel management strategies for sustainable agriculture.

## INTRODUCTION

Photosynthetic terrestrial plants play key roles as ecosystem engineers (Wright and Jones, 2006; Hartmann et al., 2009). They contribute, for instance, to the establishment of specific microbial ecological niches in plant-based systems. This is particularly the case in the rhizosphere, i.e., the soil in contact with plant roots. Besides its role in plant anchorage in soil, absorption of water and ions, nutrient storage, and plant vegetative growth, the root system is in close contact with a wide range of soil microbial populations (Berg and Smalla, 2009).

Despite their interactions with the biotic environment, the root system and its rhizosphere have received much less attention by plant physiologists than the rest of the plant. Plant roots exude a huge diversity of organic nutrients (organic acids, phytosiderophores, sugars, vitamins, amino acids, nucleosides, mucilage) and signals that attract microbial populations, especially those able to metabolize plant-exuded compounds and proliferate in this microbial habitat (Bais et al., 2006; Pothier et al., 2007; Badri et al., 2009; Shukla et al., 2011; Drogue et al., 2013). Root exudates being the largest source of carbon supply within soil, the rhizosphere compartment houses a rich microbial community, comprising up to 10^10^ bacteria per gram of soil (Gans et al., 2005; Roesch et al., 2007) and encompassing a large diversity of taxa (Kyselková et al., 2009; Gomes et al., 2010). The corresponding microbial community associated to plant roots can be referred to as the rhizo-microbiome (Chaparro et al., 2013). Its composition is distinct from that of the microbial community of the surrounding soil, a direct consequence of bacterial competition for nutrients liberated in the vicinity of plant roots (Raynaud et al., 2008; Bulgarelli et al., 2013; Chaparro et al., 2013). Since root exudate composition changes along the root system, according to stages of plant development and to plant genotypes, the rhizo-microbiome composition differs accordingly (Berg and Smalla, 2009; Aira et al., 2010; Bouffaud et al., 2012; Bulgarelli et al., 2013; Chaparro et al., 2013). Plant-driven selection of bacteria is an important issue recently discussed in several reviews (Hartmann et al., 2009; Doornbos et al., 2012; Drogue et al., 2012; Bulgarelli et al., 2013).

Within the rhizo-microbiome, some microorganisms can promote plant growth and provide better plant health through several indirect or direct mechanisms (Couillerot et al., 2009; Richardson et al., 2009). Beneficial plant-microbe interactions are symbiotic interactions in which costs and benefits are shared by the plants and the microorganisms (Odum and Barrett, 2005; Bulgarelli et al., 2013) and can be categorized into two main types of interactions (Drogue et al., 2012). First, mutualistic interactions correspond to intimate and mostly obligate interactions between microbes and a restricted range of compatible host plants. They generally lead to the formation of a structure specifically dedicated to the interaction (e.g., nodules during the symbiosis between nodulating rhizobia and Fabaceae, arbuscules in the endomycorrhizal symbiosis; Parniske, 2008; Masson-Boivin et al., 2009). Second, cooperations (also called associative symbioses) correspond to less obligate and specific interactions (Barea et al., 2005; Drogue et al., 2012). They involve soil bacteria able to colonize the surface of the root system (and sometimes root inner tissues) and to stimulate the growth and health of the plant, and are referred to as plant growth-promoting rhizobacteria (PGPR; Barea et al., 2005). Colonization of plant host roots by PGPR is heterogeneous along the root system; their competitiveness regarding this process is a *sine qua non* for plant growth promotion (discussed in Benizri et al., 2001; Compant et al., 2010; Dutta and Podile, 2010; Drogue et al., 2012). In comparison to mutualistic symbionts, PGPR are thought to interact with a large range of host plant species and to encompass a huge taxonomic diversity, especially within the Firmicutes and Proteobacteria phyla (Lugtenberg and Kamilova, 2009; Drogue et al., 2012). PGPR can enhance plant nutrition via associative nitrogen fixation, phosphate solubilization, or phytosiderophore production (Richardson et al., 2009). They can improve root development and growth through the production of phytohormones or enzymatic activities, as well as favor the establishment of rhizobial or mycorrhizal symbioses. Others can protect the plant through inhibition of phytoparasites, based on antagonism or competition mechanisms, and/or by eliciting plant defenses such as induced systemic resistance (ISR; Couillerot et al., 2009; Lugtenberg and Kamilova, 2009). Some PGPR can also help plants withstand abiotic stresses including contamination by heavy metals or other pollutants; certain are even able to increase the capacity of plants to sequester heavy metals (Jing et al., 2007; Saharan and Nehra, 2011; Tak et al., 2013). Therefore, utilizing PGPR is a new and promising approach for improving the success of phytoremediation of contaminated soils (for recent reviews see Zhuang et al., 2007; Shukla et al., 2011; Tak et al., 2013).

Understanding and quantifying the impact of PGPR on roots and the whole plant remain challenging. One strategy is to inoculate roots with a PGPR *in* *vitro* and monitor the resulting effects on plant. This showed that many PGPR may reduce the growth rate of the primary root (Dobbelaere et al., 1999), increase the number and/or length of lateral roots (Combes-Meynet et al., 2011; Chamam et al., 2013), and stimulate root hair elongation *in vitro* (Dobbelaere et al., 1999; Contesto et al., 2008). Consequently, the uptake of minerals and water, and thus the growth of the whole plant, can be increased. Some of these effects, including increased root and shoot biomass, are also documented for PGPR-inoculated plants growing in soil (El Zemrany et al., 2006; Minorsky, 2008; Veresoglou and Menexes, 2010; Walker et al., 2012).

The focus of this paper is to review the main modes of action of PGPR strains, the functioning of PGPR populations, and their ecology in the rhizosphere. Description of plant-beneficial properties of PGPR has been the focus of several reviews (e.g., Vessey, 2003; Richardson et al., 2009; Bashan and de-Bashan, 2010), but without integrating actual PGPR gene expression on roots, the interactions between different PGPR populations in the rhizosphere, or the resulting plant-beneficial effects. This paper is organized into four sections. In the first section, we present the molecular mechanisms through which PGPR may affect the architecture of the root system and interfere with the plant hormonal pathways, and review our current understanding of their impact on the structural properties of the roots. In the second section, recent findings related to the impact of PGPR on the physiology of the whole plant are presented, with a focus on plant nutrient acquisition, plant transcriptome and plant metabolome. The third section shows how expression of plant-beneficial properties can be affected within the rhizosphere by molecules emitted by other microbial populations or by the plant. As PGPR strains are not acting individually in the rhizosphere, the ecology of PGPR populations and notably the complexity of the interactions taking place between PGPR populations is discussed in the fourth section. Finally, we conclude on the importance of integrating molecular investigations on the modes of action and ecology of PGPR strains with high-throughput analyses on the abundance, taxonomic/functional diversity and activity of rhizosphere microbial communities, and with the monitoring of plant molecular responses.

## IMPACT OF PGPR ON ROOT SYSTEM ARCHITECTURE AND ROOT STRUCTURE

**FIGURE 1:**
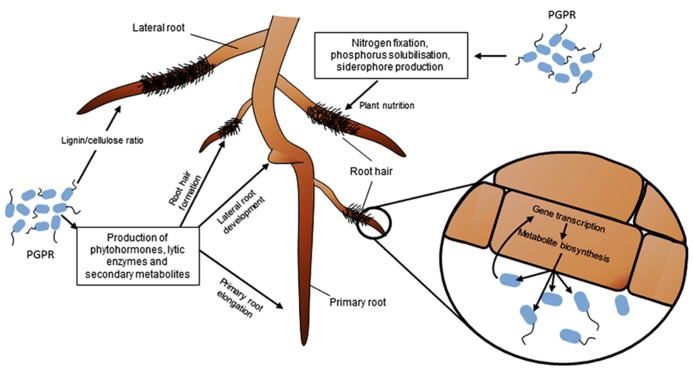
**Impact of phytostimulating PGPR on RSA, nutrient acquisition and root functioning.** PGPR can modulate root development and growth through the production of phytohormones, secondary metabolites and enzymes. The most commonly observed effects are a reduction of the growth rate of primary root, and an increase of the number and length of lateral roots and root hairs. PGPR also influence plant nutrition via nitrogen fixation, solubilization of phosphorus, or siderophore production, and modify root physiology by changing gene transcription and metabolite biosynthesis in plant cells.

Most terrestrial plants develop their root system to explore soil and find nutrients to sustain growth. Root is a complex organ made of distinct regions such as the root tip, root meristem, differentiation and elongation zones, and emerging lateral roots (Scheres et al., 2002). These regions have distinct roles. For instance, root hairs are differentiated epidermal cells important for plant mineral nutrition, as inferred from gene expression studies (Lauter et al., 1996; von Wiren et al., 2000) and nutrient accumulation measurements (Ahn et al., 2004). Root functional specificity is also reflected at the level of plant-microbe interactions. In Fabaceae for example, the root tip is the most important region to initiate the rhizobial colonization process leading eventually to the formation of a root nodule (Desbrosses and Stougaard, 2011). In Poaceae, root hairs and lateral roots are preferentially colonized by PGPR, where they may express their plant beneficial properties (Pothier et al., 2007; Combes-Meynet et al., 2011). Root system architecture (RSA) integrates root system topology, the spatial distribution of primary and lateral roots, and the number and length of various types of roots. Several abiotic and biotic factors can influence RSA, including PGPR strains. PGPR modify RSA and the structure of root tissues mainly through their ability to interfere with the plant hormonal balance (**Figure 1**).

### PGPR EFFECTS ON RSA VIA MODULATION OF HOST HORMONAL BALANCE

Changes in RSA may result from interferences of PGPR with the main hormonal pathways involved in regulating plant root development: auxin, cytokinin, ethylene, and to a lesser extend gibberellin, and abscisic acid (ABA) (Moubayidin et al., 2009; Stepanova and Alonso, 2009; Dodd et al., 2010; Overvoorde et al., 2011). The balance between auxin and cytokinin is a key regulator of plant organogenesis, and shapes root architecture (Aloni et al., 2006). The auxin to cytokinin ratio can be affected by PGPR because they are able to produce a wide range of phytohormones, including auxins and/or cytokinins, and secondary metabolites that can interfere with these hormonal pathways.

Many PGPR are able to produce phytohormones and secondary metabolites interfering with the plant auxin pathway, such as auxins, 2,4-diacetylphloroglucinol (DAPG), and nitric oxide (NO). Indole-3-acetic acid (IAA) is the best-characterized auxin produced by many plant-associated bacteria, including PGPR (Spaepen et al., 2007a). Exogenous IAA controls a wide variety of processes in plant development and plant growth: low concentrations of IAA can stimulate primary root elongation, whereas high IAA levels stimulate the formation of lateral roots, decrease primary root length and increase root hair formation (**Figure 1**; Dobbelaere et al., 1999; Patten and Glick, 2002; Perrig et al., 2007; Spaepen et al., 2007b; Remans et al., 2008). IAA is usually synthesized by rhizobacteria from tryptophan, which is found at different concentrations in root exudates according to plant genotype (Kamilova et al., 2006). In PGPR strains, several IAA biosynthetic pathways have been described depending on the metabolic intermediates (Spaepen et al., 2007a). The indole-3-pyruvate decarboxylase (encoded by the *ipdC/ppdC* bacterial gene) is a key enzyme involved in the indolepyruvic acid pathway. Effects of *ipdC* mutants on plant root morphology are often altered in comparison to those of wild-type strains (Brandl and Lindow, 1998; Dobbelaere et al., 1999; Patten and Glick, 2002; Suzuki et al., 2003; Malhotra and Srivastava, 2008).

Plant growth promotion by PGPR can also result from indirect stimulation of the plant auxin pathway. For example, several PGPR strains like *Azospirillum brasilense* have a nitrite reductase activity and consequently are able to produce NO during root colonization (Creus et al., 2005; Pothier et al., 2007; Molina-Favero et al., 2008). NO is involved in the auxin signaling pathway controlling lateral root formation (Creus et al., 2005; Lanteri et al., 2006, 2008; Molina-Favero et al., 2008). DAPG is a well-known antimicrobial compound produced by biocontrol fluorescent pseudomonads (Couillerot et al., 2009). At lower concentrations, DAPG can also be a signal molecule for plants, inducing systemic resistance (Iavicoli et al., 2003; Bakker et al., 2007), stimulating root exudation (Phillips et al., 2004), and enhancing root branching (Brazelton et al., 2008; Couillerot et al., 2011; Walker et al., 2011). DAPG can interfere with an auxin-dependent signaling pathway and thus modify RSA (Brazelton et al., 2008). Indeed, applications of exogenous DAPG, at a concentration around 10 μM, inhibited primary root growth and stimulated lateral root production in tomato seedlings. Furthermore, roots of an auxin-resistant *diageotropica* mutant of tomato displayed reduced DAPG sensitivity (Brazelton et al., 2008).

**FIGURE 2:**
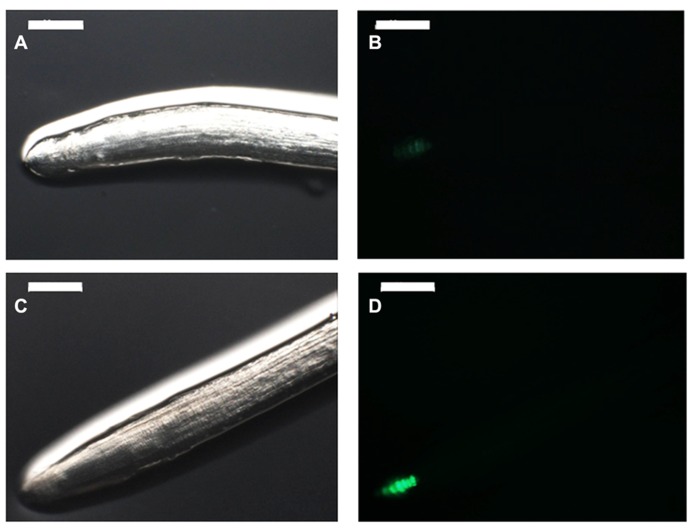
**PGPR-mediated changes in RSA may relate to modifications of auxin content in roots.** Six-day-old *Arabidopsis* plantlets expressing the *GFP* gene under the control of the auxin-sensitive *DR5* artificial promoter were inoculated **(C, D)** or not **(A, B)** with the PGPR *Phyllobacterium brassicacearum* STM196. Six days later, root tips were observed under normal light **(A, C)** or UV light **(B, D)** with a microscope (Z16APO, Leica, Bensheim, Germany). Scale bars represent 200 μm. Inoculation by STM196 modified root traits such as root hair elongation and primary root growth, which coincided with an increase in GFP signal in the root tip in inoculated **(D)** compared with control plants **(B)**. These observations confirm previous results with a different *Arabidopsis* *DR5* reporter line (Contesto et al., 2010).

The growth-promotion effect of auxin or auxin-like compounds by PGPR may require functional signaling pathways in the host plant. To test that hypothesis, one could use a host plant defective at a particular step of the hormone-signaling pathway and assess whether PGPR inoculation complements or not the effect of the mutation. This strategy requires the use of model plant such as *Arabidopsis*, the only biological system that provides to date enough documented mutant plants (Dubrovsky et al., 1994; Alonso et al., 2003). Consistent with that, some *Arabidopsis* auxin-signaling mutants failed to show the typical root architecture changes in response to the beneficial rhizobacterium *Phyllobacterium brassicacearum* STM196 (Contesto et al., 2010). However, auxin content was not increased in roots upon inoculation with *Phyllobacterium brassicacearum* STM196, ruling out the potential implication of auxin of bacterial origin (Contesto et al., 2010). Nevertheless, the use of *Arabidopsis* *DR5*::*GUS* reporter line, whose expression is restricted to the root meristem where the auxin maximum is located (Ulmasov et al., 1997; Casimiro et al., 2001), showed a change of expression pattern in response to STM196 inoculation (**Figure 2**). GUS staining appeared more intense on a wider region of the root tip as well as in the vasculature, suggesting that there was a change of auxin distribution in the root in response to STM196 inoculation, even though this strain is a low auxin producer (Contesto et al., 2010). Interestingly, a similar observation was made when *Arabidopsis* plantlets were inoculated with the PGPR *Bacillus* *subtilis* GB03 (Zhang et al., 2007), which emits volatile organic compounds (VOCs), or with *Pseudomonas fluorescens* WCS417 (Zamioudis et al., 2013).

Cytokinin production (especially zeatin) has been documented in various PGPR like *Arthrobacter giacomelloi*, *Azospirillum brasilense, Bradyrhizobium japonicum, Bacillus licheniformis, Pseudomonas fluorescens*, and *Paenibacillus polymyxa* (Cacciari et al., 1989; Timmusk et al., 1999; de García Salamone et al., 2001; Perrig et al., 2007; Cassán et al., 2009; Hussain and Hasnain, 2009). Cytokinins stimulate plant cell division, control root meristem differentiation, induce proliferation of root hairs, but inhibit lateral root formation and primary root elongation (Silverman et al., 1998; Riefler et al., 2006). Inoculation of plants with bacteria producing cytokinin has been shown to stimulate shoot growth and reduce the root to shoot ratio (Arkhipova et al., 2007). Bacterial genes involved in cytokinin biosynthetic pathways have been identified *in silico* but their role has not yet been validated through functional analyses (Frébort et al., 2011). Consequently, the contribution of cytokinin production by PGPR to RSA modifications remains speculative.

Ethylene is another key phytohormone, which inhibits root elongation and auxin transport, promotes senescence and abscission of various organs, and leads to fruit ripening (Bleecker and Kende, 2000; Glick et al., 2007). Ethylene is also involved in plant defense pathways (Glick et al., 2007). This phytohormone can be produced in small amounts from the precursor methionine by some PGPR, like *Azospirillum brasilense* (Thuler et al., 2003; Perrig et al., 2007). The ability of *Azospirillum brasilense* to produce ethylene presumably promotes root hair development in tomato plants. Indeed, exogenous ethylene supply to the plant mimicked the effect of *Azospirillum brasilense* inoculation, while the addition of an ethylene biosynthesis inhibitor blocked this effect (Ribaudo et al., 2006). Actually, PGPR are more widely able to lower plant ethylene levels through deamination of 1-aminocyclopropane-1-carboxylic acid (ACC). Many genomes of PGPR do contain a gene (*acdS*) coding for an ACC deaminase, which degrades ACC into ammonium and α-ketobutyrate (Blaha et al., 2006; Contesto et al., 2008; Prigent-Combaret et al., 2008). By lowering the abundance of the ethylene precursor ACC, the PGPR AcdS activity is thought to decrease root ethylene production, which can in turn alleviate the repressing effect of ethylene on root growth (Glick, 2005). Despite being widely accepted and supported by experimental data (Penrose et al., 2001; Contesto et al., 2008), the model raises issues that have not been well addressed yet. The first one deals with ethylene production within roots. Light is promoting ethylene biosynthesis, providing there is a sufficient CO_2_ supply for shoots (Yang and Hoffman, 1984). Exposition of roots to light was shown to trigger an increase in ethylene production (Lee and Larue, 1992). In soil however, roots are sheltered from light, suggesting that this organ might not be able to synthesize large amounts of ethylene. In agreement with that, Fabaceae roots did produce ethylene in response to rhizobial colonization in presence of light, but less when they were in the dark (Lee and Larue, 1992). Secondly, there is a regulation of ethylene synthesis by a feedback loop (Yang and Hoffman, 1984). This loop should stimulate ethylene biosynthesis when the level of ACC is low. Unless PGPR disconnect that feedback loop, lowering ACC content would eventually result in stimulation of ethylene production. There is no indication yet how the feedback loop would work in presence of a PGPR. Last but not least, if ethylene plays a key role during the plant-PGPR interaction, one would expect that either plant ethylene mutants or impaired AcdS activity in the bacteria would result in clear disturbance of the plant responses to bacteria. However, minor effects on RSA were observed when plants were inoculated with an *acdS* bacterial mutant, or when plants affected in their ethylene signaling pathway were inoculated with wild-type PGPR (Contesto et al., 2008; Galland et al., 2012; Zamioudis et al., 2013). It suggests that ethylene participates to the root architecture response but is not a key player. Taken together, the functional importance of the bacterial ACC deaminase function needs further clarification. One hypothesis could be that AcdS contributes to the fine-tuning of ethylene biosynthesis during the plant-PGPR cooperation.

Several reports have revealed that PGPR are able to produce ABA or gibberellic acid, or to control the level of these hormones in plants (Richardson et al., 2009; Dodd et al., 2010). The first one, ABA, is well known for its involvement in drought stress. During water stress, increase in ABA levels causes closing of stomata, thereby limiting water loss (Bauer et al., 2013). However, this hormone also plays different roles during lateral root development (De Smet et al., 2006; Dodd et al., 2010). Inoculation with *Azospirillum brasilense* Sp245 led to an increase of ABA content in *Arabidopsis*, especially when grown under osmotic stress (Cohen et al., 2008). Gibberellins promote primary root elongation and lateral root extension (Yaxley et al., 2001). Production of gibberellins has been documented in several PGPR belonging to *Achromobacter xylosoxidans, Acinetobacter calcoaceticus, Azospirillum *spp.*, Azotobacter *spp., *Bacillus *spp*.*, *Herbaspirillum seropedicae, Gluconobacter diazotrophicus* and rhizobia (Gutiérrez-Mañero et al., 2001; Bottini et al., 2004; Dodd et al., 2010). Application of gibberellic acid on maize, at a concentration similar to that produced by *Azospirillum*, promotes root growth; furthermore, gibberellin content increases in maize root inoculated with *Azospirillum* (Fulchieri et al., 1993). In addition to playing a role in plant RSA, these two hormones are involved in plant defense mechanisms. Thus, PGPR producing these hormones may modulate the hormonal balance involved in plant defense, including the jasmonate and salicylic acid pathways (for a review see Pieterse et al., 2009).

Although the production of hormones by PGPR has been well described, the genetic determinants involved in their biosynthesis remain largely unknown and bacterial mutants affected in hormone biosynthesis are mostly lacking. Consequently, the involvement of hormones of bacterial origin in the modulation of plant hormonal balance has not been fully demonstrated.

### MODIFICATION OF ROOT CELL WALL AND ROOT TISSUE STRUCTURAL PROPERTIES BY PGPR

Many PGPR can lead to modifications of the chemical composition and therefore structural properties of root cell walls (**Figure 1**; El Zemrany et al., 2007; Zhang et al., 2007). For example, the biocontrol agent *Bacillus pumilus* INR-7 is able to enhance lignin deposition in pearl millet epidermal tissues, and this plant defense response appears much more rapidly in PGPR-primed plants infected by the pathogen *Sclerospora graminicola* compared to non-primed plants (Niranjan Raj et al., 2012). The sole inoculation of INR-7 led to callose apposition. Although the precise location of these deposited polymers was not investigated, it is possible that their enhanced accumulation may participate to pathogen inhibition and disease suppression. A similar response was also triggered by *Bacillus pumilus* SE34 and *Bacillus subtilis* UMAF6639 when inoculated to respectively pea and melon roots. In both cases, it led to enhanced fungal pathogen tolerance (García-Gutiérrez et al., 2013). Inoculation of *Pseudomonas fluorescens* 63-28R to pea roots induced accumulation of lignin in root cells and inhibited colonization by the oomycete *Pythium ultimum* (Benhamou et al., 1996). The same result was observed with a *Pseudomonas putida* strain inoculated on bean roots (Anderson and Guerra, 1985). These cell wall modifications have been reported in the case of PGPR that protect plants against phytopathogens by activating ISR plant defense responses (Iavicoli et al., 2003; Desoignies et al., 2012; Weller et al., 2012; García-Gutiérrez et al., 2013). One of the consequences of ISR is thus the reinforcement of the cell wall through enhanced lignin synthesis and callose apposition (Kovats et al., 1991; Strömberg and Brishammar, 1993), which restricts the progression of phytopathogens through plant tissues (García-Gutiérrez et al., 2013).

Modifications of the chemical composition of root cell walls are also triggered by PGPR that directly promote plant growth (**Figure 1**). Through the analysis of the infrared spectral characteristics of crude cell wall preparations of maize roots, El Zemrany et al. (2007) concluded that roots inoculated with *Azospirillum lipoferum* CRT1 had lower lignin content than uninoculated ones. This result contrasts with those aforementioned for biocontrol agents. Nevertheless, lower lignin content may facilitate cell elongation, and therefore overall root elongation. Similarly, *Azospirillum irakense* produces pectate lyases that are capable of degrading the pectate content of root cell wall and might allow its progression between root cortex cells and its functioning as an endophyte (Bekri et al., 1999). Up to now, the impact on plant lignin content of PGPR that are both inducing ISR and promoting root growth has not been clarified.

Modifications of root cell wall ultrastructure are thought to result mainly from PGPR-triggered changes in plant gene expression. Indeed, *Bacillus subtilis* GB03 promotes *Arabidopsis* growth by producing VOCs that were shown to modulate the expression of 38 genes with known functions associated with cell wall structure (Ryu et al., 2003; Zhang et al., 2007). Among them, 30 were implicated in cell wall expansion or loosening. The endophytic PGPR *Azospirillum irakense* was also shown to stimulate the expression of polygalacturonase genes in inoculated rice roots (Sekar et al., 2000).

Chemical mediators involved in the effects of PGPR on root cell walls have received little attention. A single report has indicated that the exogenous addition of auxins enhanced the induced polygalacturonase activities observed in *Azospirillum irakense *inoculated rice roots (Sekar et al., 2000).

## SYSTEMIC EFFECTS OF PGPR ON WHOLE PLANT PHYSIOLOGY AND FUNCTIONING

In addition to their effects on root tissues, PGPR can modify the physiology and functioning of plant tissues located at a substantial distance from the colonized sites, such as shoots. Two types of mechanisms are involved. On the one hand, some PGPR can enhance nutrient availability/uptake for plant roots. Stimulation of plant nutrition will lead to modifications in primary metabolism and consequently will contribute to enhance growth. On the other hand, certain PGPR trigger specific systemic responses, mostly by unknown signaling mechanisms. High-throughput analyses of plant transcriptomic and metabolomic responses have evidenced the effects of PGPR on plant gene expression and metabolite accumulation, respectively. These results highlight the extensive effect of PGPR on whole plant physiology and functioning (**Figure 1**), and provide clues to understand the systemic effect of PGPR.

### IMPACT OF PGPR ON PLANT NUTRITION

The impact of PGPR on plant nutrition may result from effects on plant nutrient uptake and/or on plant growth rate (Mantelin and Touraine, 2004). It is indeed commonly hypothesized that nutrient uptake is increased as a consequence of increased root surface area triggered by PGPR. However, root ion transporters are under the control of regulatory processes that adjust their activity to the plant nutritional demand (Imsande and Touraine, 1994; Lappartient and Touraine, 1996; Lappartient et al., 1999; Nazoa et al., 2003), so that regulations of root development and ion transporter activities are antagonistically coordinated to maintain steady nutrient acquisition rate (Touraine, 2004). Hence, PGPR must interfere with pathways coordinating plant development and plant nutrition to elicit both increased nutrient acquisition rate and plant growth promotion (**Figure 1**).

Plant growth-promoting rhizobacteria can directly increase nutrient supply in the rhizosphere and/or stimulate ion transport systems in root. With regards to increased nutrient supply, two main types of bacterial activities can be considered. Firstly, phosphate solubilization is one key effect of PGPR on plant nutrition. Soils generally contain a large amount of phosphorus, which accumulates in the wake of regular fertilizer applications, but only a small proportion of the latter is available for plants. Plants are able to absorb on their own mono and dibasic phosphate; organic or insoluble forms of phosphate need to be mineralized or solubilized by microorganisms, respectively (Richardson et al., 2009; Ramaekers et al., 2010). Many PGPR – such as *Pseudomonas*, *Bacillus*, *Rhizobium *– are able to dissolve insoluble forms of phosphate (for a review see Richardson et al., 2009). Two main processes exist: acidification of the external medium through the release of low molecular weight organic acids (such as gluconic acid) that chelate the cations bound to phosphate (Miller et al., 2009), and production of phosphatases/phytases that hydrolyse organic forms of phosphate compounds. Secondly, many associated bacteria can fix N_2_ so that they could provide nitrogen to the plant. Evidence in favor of the participation of PGPR to the plant N budget has been reported for several plants, especially sugar cane (Boddey et al., 2003). However, the impact of N_2_-fixation by PGPR is still debated and is rarely credited for the stimulation of plant growth (for review see Dobbelaere et al., 2003). In addition, non-fixing rhizobacteria can promote plant growth, showing that N provision is not obligatory for plant growth promotion. For instance, *Phyllobacterium brassicacearum* STM196 is unlikely to fix N_2_ while it promotes the growth of canola and *Arabidopsis* (Bertrand et al., 2000, 2001; Mantelin et al., 2006).

With regards to the impact of PGPR on nutrient uptake systems, only very few studies have been published so far. Inoculation of canola with *Achromobacter* sp. strain U80417 resulted in an increase of both ${NO}_{3}^{-}$ and K^+^ net influx rates per root surface area unit (Bertrand et al., 2000). In this study, the net H^+^ efflux was also enhanced, so that increased ${NO}_{3}^{-}$ and K^+^ uptake rates may be part of a general mechanism leading to increased ion uptake rate, similar to energization of nutrient transport by enhanced proton pump activity (Sondergaard et al., 2004). In favor of this hypothesis, acidification of the rhizosphere has also been reported with *Arabidopsis* exposed to the VOC-emitting *Bacillus subtilis* strain GB03 (Zhang et al., 2009).

In *Arabidopsis*, ${NO}_{3}^{-}$ uptake measurement in response to PGPR, over time, can lead to contradictory results: ${NO}_{3}^{-}$ influx was increased in seedlings, upon 24 h-inoculation with *Phyllobacterium brassicacearum* STM196, while it was reduced 7 days later (Mantelin et al., 2006). However, it is hard to draw a firm conclusion as the net ${NO}_{3}^{-}$ uptake rate remained unknown since ion efflux was not measured in these experiments. Except for the *NRT2.5* and *NRT2.6* genes, the accumulation of transcripts of nitrate and ammonium transporters was very slightly or not significantly changed upon *Phyllobacterium brassicacearum* STM196 inoculation (Mantelin et al., 2006). Interestingly, these two genes have recently been shown to be required in *Arabidopsis* growth promotion by this PGPR (Kechid et al., 2013). Since these two genes code for two plasma membrane-localized ${NO}_{3}^{-}$ transporters (Kotur et al., 2012), this discovery raises the question of the interactions between N nutrition and plant development in PGPR-inoculated plants. The *NRT2.5* and *NRT2.6* genes are predominantly expressed in shoots (Mantelin et al., 2006). Their role in *Phyllobacterium brassicacearum* STM196 plant growth promotion and/or root architecture modification are not linked to changes in ${NO}_{3}^{-}$ uptake rate or ${NO}_{3}^{-}$ distribution between roots and shoots (Kechid et al., 2013), suggesting an involvement in N-signaling rather than a direct role in N-metabolism.

Evidence in favor of a regulation of ion transporters at a transcriptional level by PGPR has been obtained in studies with *Bacillus subtilis* GB03. This strain induces concomitant down- and up-regulation of *HKT1 *expression in roots and shoots of *Arabidopsis* seedlings, respectively (Zhang et al., 2008). In the shoots, HKT1 functions in phloem tissues to retrieve Na^+^ from the xylem (Berthomieu et al., 2003) and in the roots it is involved in Na^+^ uptake (Rus et al., 2001). The differential regulation of *HKT1 *expression in roots and shoots resulted in reduced accumulation of Na^+^ and increased accumulation of K^+^ in both organs of GB03-inoculated seedlings under salt-stress conditions (Zhang et al., 2008). Consistent with the effect of GB03 on HKT1, GB03 failed to rescue salt-stressed *hkt1 *mutant seedlings from elevated Na^+^ accumulation.

Volatile organic compounds emitted by GB03 also activate the plant’s iron acquisition machinery leading to increased iron assimilation (Zhang et al., 2009). Firstly, this PGPR leads to acidification of the rhizosphere, both directly due to chemical effects of some unidentified VOCs and indirectly through increased root proton efflux. Secondly, GB03 up-regulates the expression levels of *FRO2* and *IRT1* genes, coding respectively for a Fe^3^^+^ chelate reductase and a Fe^2^^+^ transporter. As a result, GB03-exposed *Arabidopsis* has enhanced ferric chelate reductase activity and increased iron content. Finally, it has been shown that this PGPR induces the expression of the *FIT1* transcription factor that regulates positively *FRO2* and *IRT1* expressions (Zhang et al., 2009). The fact that GB03 fails to increase root ferric reductase activity and plant iron content in *Arabidopsis* *fit1* mutants shows that PGPR can modify indirectly ion uptake by interfering with plant regulatory processes that control ion transporter expressions and/or activities (Zhang et al., 2009).

### IMPACT OF PGPR ON PLANT TRANSCRIPTOME

Targeted or genome-wide analyses of plant gene expression following root inoculation by PGPR were reported with various bacterial models: phytostimulating PGPR, endophytes and PGPR exerting a biocontrol activity. Inoculation of the phytostimulator *Pseudomonas putida* MTCC5279 triggered overexpression of 520 genes and repression of 364 genes (threefold changes) in leaves of *Arabidopsis*; upregulated genes were involved in maintenance of genome integrity, growth hormone and amino acid syntheses, ABA signaling and ethylene suppression, Ca^2^^+^ dependent signaling and induction of ISR (Srivastava et al., 2012). On rice, a recent study performed with *Azospirillum* points towards association specificity (Vargas et al., 2012). The targeted expression of ethylene receptors was followed after inoculation of *Azospirillum brasilense* Sp245 on two rice cultivars of contrasted ability to gain nitrogen from biological nitrogen fixation. Seedlings of cultivar IR42, which enabled higher nitrogen fixation, also displayed higher expression of ethylene receptors compared to cultivar IAC 4440 (Vargas et al., 2012). The transcript accumulation of all ethylene receptors might be necessary for the establishment of a beneficial association between the plant and the bacteria.

As for endophytes, differential colonization of rice roots was observed with an *Azoarcus* PGPR. In a less compatible interaction, a slight defense response occurred and was accompanied by the induction of pathogenesis-related proteins and proteins sharing domains with receptor-like kinases induced by pathogens; those proteins were also induced by a jasmonate treatment (Miché et al., 2006). Inoculation of rice roots with the endophytic PGPR *Herbaspirillum seropedicae* triggered the expression of genes responsive to auxin and ethylene and the repression of the defense-related proteins PBZ1 and thionins (Brusamarello-Santos et al., 2012). These studies suggest that endophytes modulate plant defense responses during colonization.

Plants treated with biocontrol PGPR, usually belonging to the *Pseudomonas* genus, are more resistant to subsequent infections by bacterial or fungal pathogens. In *Arabidopsis*, this rhizobacteria-mediated ISR requires sensitivity to jasmonate and ethylene, and the regulators MYC2 (Pieterse et al., 1996, 2000; Pozo et al., 2008), NPR1 (Pieterse et al., 1998), and MYB72 (Van der Ent et al., 2008) played a central role in this signaling. One of the earliest transcriptomic study performed with *Pseudomonas fluorescens* WCS417r indicated that bacteria elicited a substantial change in the expression of 97 genes in roots whereas none of the approximately 8,000 genes tested showed a consistent change in expression in the leaves (Verhagen et al., 2004). Subsequent studies on *Arabidopsis* reported an increase of defense-related transcripts, including PR-related proteins, in shoots of bacterized plants compared to untreated shoots (Cartieaux et al., 2003; Wang et al., 2005; van de Mortel et al., 2012). Interestingly, the ISR induced by *Pseudomonas fluorescens* SS101 was recently reported to be dependent on salicylic acid signaling and not on jasmonic acid and ethylene signaling (van de Mortel et al., 2012); moreover, a prominent role of camalexin and glucosinolates in the ISR was proposed. In wheat, bacterization with *Pseudomonas fluorescens* Q8r1-96 also triggered the accumulation of defense-related transcripts (Okubara et al., 2010; Maketon et al., 2012) and neither DAPG nor the type three secretion system were key single factors in the expression of these genes (Maketon et al., 2012). The establishment of beneficial associations requires mutual recognition and substantial coordination of plant and microbial responses and consequently beneficial microbes modulate plant immunity.****

### IMPACT OF PGPR ON PLANT METABOLOME

Several studies have addressed the metabolomic changes triggered by PGPR inoculation, by analyzing metabolite contents of root exudates, root tissues and shoots under normal or stressful conditions (**Figure 1**). Some studies have shown that PGPR can elicit changes in the activities of root enzymes involved in the production of metabolites, especially flavonoids, leading to changes in the pattern of root exudation (Lavania et al., 2006; Shaw et al., 2006). Some *Azospirillum* PGPR stimulated by up to one-third the level of carbon compounds exuded from roots (Heulin et al., 1987). Moreover, compounds of microbial origin, such as phenazines and DAPG, could enhance total net efflux of amino acids in plant species (Phillips et al., 2004). On soybean roots, the PGPR *Chryseobacterium balustinum* Aur9 influences flavonoids exudation (Dardanelli et al., 2010). PGPR strains from *Chryseobacterium *(Dardanelli et al., 2010) or *Azospirillum* (Burdman et al., 1996) may influence flavonoid exudation by Fabacea roots. This property can be important for the design of mixed inoculants that will include a PGPR strain promoting flavonoid exudation together with rhizobia that will respond to plant flavonoids (Burdman et al., 1996).

In addition to effects on root exudates, PGPR can trigger modifications of metabolite composition of the whole plant. For instance, rice plants inoculated with *Herbaspirillum seropedicae* showed higher shoot contents in malate and in key amino acids than those of control plants (Curzi et al., 2008). Many more studies focused on modifications of secondary metabolites. Elicitation of isoflavone accumulation was observed on soybean inoculated with various PGPR, either by increasing the total isoflavone content in seedlings or by causing an asymmetric distribution of isoflavones throughout the plant (Ramos-Solano et al., 2010). Increase in the content of several alkaloid and terpenoid compounds of pharmaceutical relevance was demonstrated in medicinal plants following PGPR inoculation (Manero et al., 2003; Jaleel et al., 2007; Bharti et al., 2013). Recent studies investigated the early impact of several *Azospirillum* strains on root and shoot secondary metabolite profiles of maize and rice; analysis of secondary metabolites of two maize cultivars, inoculated by three different *Azospirillum* strains under greenhouse conditions, revealed major qualitative and quantitative modifications of the contents of secondary metabolites, especially benzoxazinoids (Walker et al., 2011). In the same way, a metabolic profiling approach of two rice cultivars inoculated with two different *Azospirillum* strains under gnotobiotic conditions, showed that profiles of secondary metabolites were modified with phenolic compounds such as flavonoids and hydroxycinnamic derivatives being the main metabolites affected (Chamam et al., 2013). Both studies evidenced a specific response, as plant metabolic changes differed according to the *Azospirillum* strain-cultivar combination. Moreover, PGPR applied to the roots can affect the composition of secondary metabolites in shoots, pointing towards systemic effects (Chamam et al., 2013).

Accumulation of secondary compounds was also modified in several plants inoculated with consortia containing arbuscular mycorrhizal fungus and PGPR. Blumenin accumulation triggered by *Rhizophagus irregularis* (formerly *Glomus intraradices*) in barley and wheat roots was increased when a rhizosphere bacterium was applied with the fungus (Fester et al., 1999). Leaf secondary metabolites (total phenols and ortho dihydroxy phenols), as well as leaf mineral content (phosphorus, potassium, zinc, copper, and iron) were maximal when *Begonia malabarica* or *Solanum viarum* were inoculated with consortia containing two fungi and a *Bacillus coagulans* strain (Selvaraj et al., 2008; Hemashenpagam and Selvaraj, 2011). Field-inoculation of maize with selected strains of *Pseudomonas*, *Azospirillum *or *Rhizophagus*/*Glomus*, or with these strains combined two by two or all three together, led to qualitative and quantitative modifications of root secondary metabolites, particularly benzoxazinoids and diethylphthalate (Walker et al., 2012). These modifications depended on fertilization level and on the type of microorganisms inoculated. The three selected strains gave distinct results when used alone, but unexpectedly all microbial consortia gave somewhat similar metabolic responses.

Plant growth-promoting rhizobacteria can help plants to withstand saline stress, a feature that may be linked to accumulation of specific metabolites. A higher level of proline content was reported in inoculated *Bacopa monnieri* (Bharti et al., 2013), as well as higher accumulation of glycine betaine-like quaternary compounds in rice inoculated with *Pseudomonas pseudoalcaligenes* (Jha et al., 2011). Similarly, *Arabidopsis* inoculation with the VOC-emitting strain *Bacillus subtilis* GB03 induced strong plant accumulation of glycine betaine and its precursor choline, and GB03-induced drought tolerance was lost in the *xipotl* mutant of *Arabidopsis* with reduced choline production (Zhang et al., 2010). Alleviation of cold stress was demonstrated for *Burkholderia phytofirmans* PsJN on grapevine; this endophytic strain promotes plant post-chilling recovery by improving acclimation to cold (Ait Barka et al., 2006). This is accompanied by accumulation of stress-related metabolites such as proline, malondialdehyde and aldehydes (known as lipid peroxidation markers), hydrogen peroxide, and by higher expression of defense- and cold-related genes (Theocharis et al., 2012). Bacterization resulted in a 1.2-fold increase in starch content and in a two-fold increase in total soluble sugars, with sugars known to be involved in low-temperature tolerance (glucose, sucrose, and raffinose) displaying higher concentrations in treated plantlets (Fernandez et al., 2012). Independently of temperature, inoculation also enhanced phenolic content (Ait Barka et al., 2006).

## EXPRESSION OF PLANT-BENEFICIAL FUNCTIONS OF PGPR IN THE RHIZOSPHERE

One PGPR strain can harbor several plant-beneficial properties, which may be co-regulated or not. Within the rhizosphere, the expression of PGPR’s plant-beneficial properties is affected by both abiotic factors (like pH, oxygen, clay mineralogy, heavy metals, etc.) and biotic factors (i.e., compounds produced by plants or the rhizo-microbiome) that can lead to distinct expression patterns in space and time, possibly with different effects on host plant (Piccoli and Bottini, 1994; Pothier et al., 2008; Prigent-Combaret et al., 2008; Dutta and Podile, 2010; Almario et al., 2013b; Drogue et al., 2013). In this section, a focus is put on the regulation of the expression of PGPR plant-beneficial properties by biotic factors occurring in the rhizosphere.

### REGULATION OF PGPR FUNCTIONS BY ROOT EXUDATES

Through the release of root exudates, plants can impact bacterial gene expression, especially genes encoding plant-beneficial traits. Composition of root exudates is dependent upon intra and inter-specific genetic variability (Bertin et al., 2003; Czarnota et al., 2003; Phillips et al., 2004), plant developmental stage (Lynch and Whipps, 1990; Bacilio-Jiménez et al., 2003) and soil abiotic factors (Lipton et al., 1987). One of the major studies aiming at analyzing the impact of root exudates variability on bacterial gene expression was carried out on *phlA*, involved in DAPG biosynthesis, in *Pseudomonas protegens *(formely* Pseudomonas fluorescens*) CHA0 (Notz et al., 2001). The expression of *phlA* was increased fourfold in the rhizosphere of monocots (maize and wheat) compared to the rhizosphere of dicots (bean and cucumber). The analysis of six maize cultivars also revealed that *phl* expression and hence biocontrol activity could be affected by plant genotype (Notz et al., 2001). Specific components of root exudates, notably sugars, were shown to affect the production of antimicrobial compounds, such as DAPG, pyoluteorin and pyrrolnitrin by fluorescent pseudomonads, with some strain-dependent effects (Duffy and Défago, 1999). Among 63 plant compounds related to defense or development, or involved in plant-microbe interactions (flavonoids, phenolic acids, phytohormones, etc.), many could modulate the expression of *phlA* and *pltA* in *Pseudomonas protegens *CHA0 (de Werra et al., 2011). No specific chemical structures were identified that generally induced or repressed *phlA* or *pltA* expression (de Werra et al., 2011). Umbelliferone led to the strongest inhibition of *phlA*; salicylate, jasmonate, and methyl jasmonate, all slightly reduced *phlA* expression, whereas the plant hormone IAA induced *phlA* expression. None of these compounds had an effect on *pltA* expression (de Werra et al., 2011) whereas a previous study reported repression of both DAPG and pyoluteorin biosynthesis genes by salicylate (Baehler et al., 2005).

1-Aminocyclopropane-1-carboxylic acid deamination (encoded by *acdS*) is another bacterial function that may be differentially expressed according to plant genotypes. Indeed, *in vitro* experiments demonstrated that some compounds present in root exudates tightly control *acdS* expression. First, ACC, the precursor of ethylene that is metabolized by AcdS, can positively regulate *acdS* expression (Hontzeas et al., 2004; Prigent-Combaret et al., 2008). Second, leucine, by inhibiting oligomerization of the Lrp-type regulator AcdR, prevents transcription of *acdS* leading to a decrease of ACC deaminase activity in *Pseudomonas putida* UW4 (Li and Glick, 2001) and in *Azospirillum lipoferum *4B (Prigent-Combaret et al., 2008). Finally, carbon sources can also influence *acdS* transcription (Prigent-Combaret et al., 2008).

As presented above, bacterial IAA biosynthesis mostly depends on tryptophan-related pathways (Spaepen et al., 2007a). The main source of tryptophan for PGPR is root exudates. Measurement of tryptophan bioavailability from graminaceous roots (*Avena barbata*) indicated that this amino acid is abundant at the emergence of secondary roots (Jaeger et al., 1999). In the absence of exogenous tryptophan supply, bacterial IAA biosynthesis is insignificant (Ona et al., 2005; Malhotra and Srivastava, 2006). Next to being an IAA precursor, tryptophan also plays an important role in regulating positively the *ipdC/ppdC* gene (Ona et al., 2005). Other root-exuded amino acids like tyrosine and phenylalanine can also induce *ipdC/ppdC *expression (Rothballer et al., 2005). Besides amino acids, plant roots release compounds like vitamins (e.g., pyridoxine and nicotinic acid) and organic acids (e.g., phenylacetic acid and prephenic acid; Shukla et al., 2011). All these compounds increase significantly IAA production in *Azospirillum brasilense *Sp245 (Zakharova et al., 2000; Somers et al., 2005).

Metabolites present in root exudates can thus specifically modulate the expression of key genes involved in plant-beneficial functions. Consequently, specific physiological responses of the plant are dependent on the PGPR strain/plant cultivar combination (Drogue et al., 2012).

### REGULATION OF PGPR FUNCTIONS BY MICROBIAL SIGNALS

Plant growth-promoting rhizobacteria exchange several types of cell-to-cell communication signals between each other and with other rhizosphere-inhabiting bacteria and fungi, i.e., quorum-sensing (QS) signals that allow bacteria to monitor their density and to coordinate gene expression only when a quorum of cells is achieved (Fuqua et al., 1994) and other bacterial signals that regulate gene expression independently of the cell density.

Quorum-sensing relies on the synthesis and perception of small diffusible molecules, such as *N*-acyl-homoserine lactones (AHLs). In fluorescent pseudomonads, colonization properties and biosynthesis of antimicrobial metabolites, such as phenazines, is often subjected to an AHL-based QS regulation (Pierson et al., 1994; Chin-A-Woeng et al., 2001; De Maeyer et al., 2011). Production of pyrrolnitrin in *Serratia plymuthica *HRO-C48, a strain isolated from the rhizosphere of oilseed rape and able to protect crops against *Verticillium* wilt, is also under QS regulation (Liu et al., 2007). In *S. plymuthica *G3, an endophytic strain, QS positively regulates antifungal activity, production of exoenzymes, but negatively regulates IAA production (Liu et al., 2011). Among the genus *Azospirillum*, only a few strains belonging to the *lipoferum* species and isolated from rice, display the ability to produce AHL signals (Vial et al., 2006). In the rice endophyte *Azospirillum lipoferum* B518, AHL inactivation abolishes pectinase activity, increases siderophore synthesis and reduces IAA production (in stationary phase) but no effect is observed on cellulase activity and on the phytostimulatory effect (Boyer et al., 2008). Moreover, a proteomic approach indicates that AHL-based QS regulation in *Azospirillum* is rather dedicated to control functions linked to rhizosphere competence and adaptation to plant roots (Boyer et al., 2008).

Interestingly, several studies have shown that bacterial communication of a specific bacterial population could be jammed by other microbes; indeed, some soil bacteria can inactivate AHL (notably members of the genus *Bacillus*), whereas others can intercept AHL or can act as a physical barrier preventing their diffusion (Boyer and Wisniewski-Dyé, 2009). Consequently, other members of the bacterial rhizosphere community can compromise expression of biocontrol traits in PGPR. Conversely, cross-talk between species using the same AHL signal or a structurally-related AHL can occur in natural habitats and was evidenced in the rhizosphere of wheat and tomato (Pierson et al., 1998; Steidle et al., 2001). Finally, plant compounds designated as AHL-mimics can also interfere with bacterial QS and may influence the expression of plant-beneficial functions (Teplitski et al., 2000; Vandeputte et al., 2010). Some *Pseudomonas fluorescens* strains unable to synthetize AHLs but possessing the cognate receptor may even recognize a plant compound to trigger expression of genes involved in biocontrol properties (Subramoni et al., 2011).

Exometabolites produced by microbial populations including pathogenic fungal strains can also affect PGPR plant-beneficial properties. For instance, fusaric acid produced by *Fusarium oxysporum* represses the production of DAPG in the biocontrol strain *Pseudomonas protegens* CHA0 (Notz et al., 2002). Next to their antifungal effect, some *Pseudomonas*-produced compounds can influence gene expression of biocontrol traits in pseudomonads. Indeed, in *Pseudomonas protegens *strains CHA0 and Pf-5, DAPG and pyoluteorin productions are influenced by positive autoregulation; moreover, DAPG and pyoluteorin mutually inhibit one another’s production (Brodhagen et al., 2004; Baehler et al., 2005). In order to determine if DAPG could act as a signal on other PGPR strains than those of the fluorescent *Pseudomonas* group, a differential fluorescence induction promoter-trapping approach based on flow cytometry was developed on *Azospirillum*. Using this approach DAPG was shown to enhance expression of a wide range of *Azospirillum brasilense* genes, including genes involved in phytostimulation. Four of them (i.e., *ppdC*, *flgE*, *nirK*, and *nifX-nifB*) were upregulated on roots in the presence of *Pseudomonas fluorescens* F113 compared with its DAPG-negative mutant (Combes-Meynet et al., 2011). Accordingly, *Pseudomonas fluorescens* F113 but not its DAPG-negative mutant enhanced the phytostimulatory effect of *Azospirillum brasilense* Sp245 on wheat. Thus, DAPG can act as a signal by which some beneficial pseudomonads may stimulate plant-beneficial activities of *Azospirillum* PGPR (Combes-Meynet et al., 2011). This finding is also relevant in the context of inoculation with microbial consortia, in which different types of PGPR may be combined. The number of studies investigating the efficacy of such combined inoculations is growing, with variations in the number of microorganisms and the nature of the combinations (PGPR strains only, PGPR and fungi, etc.; Cassán et al., 2009; Couillerot et al., 2012; Kumar et al., 2012; Walker et al., 2012). Field inoculation of sorghum with fluorescent *Pseudomonas* strains alone or in combination with arbuscular mycorrhizal fungi showed a better effect when in presence of the latter (Kumar et al., 2012). Field inoculation of maize with a consortium consisting of two PGPR (*Azospirillum lipoferum* CRT1 and *Pseudomonas fluorescens* F113) and one mycorrhizal strain (*Rhizophagus irregularis*/*Glomus intraradices *JJ291) showed an increase of root surface, root volume and number of roots, although data were not statistically significant compared to the single *Rhizophagus* inoculation (Walker et al., 2012). Modification of one member of this consortium (three different *Azospirillum* strains were tested) could lead to significant modification of maize growth (Couillerot et al., 2012). Further studies are needed to describe the synergistic effects between beneficial microorganisms at a molecular scale and to analyse the expression of plant-beneficial functions when consortia are used.

## ECOLOGY OF PGPR POPULATIONS AND IMPACT ON ROOT SYSTEM FUNCTIONING

**FIGURE 3:**
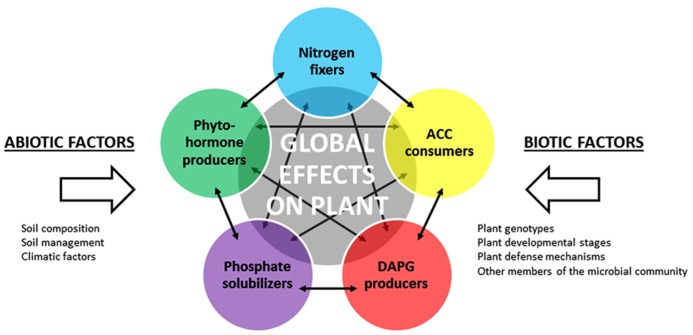
**Implementation of plant-growth promoting traits in PGPR functional groups.** Selected PGPR functional groups are represented by different colored circles. The resulting effect of all PGPR functional groups on the plant is symbolized by the gray circle. Abiotic and biotic factors may influence the activity of each functional group. Solid arrows represent potential interactions (inhibition, signaling, etc.) between members of the functional groups, which may impact on the size, diversity and activity of these groups.

Many studies have deciphered the mechanisms of action of PGPR using one individual strain and one host plant. But in reality, as described above, PGPR strains are not acting individually in the rhizosphere but rather as part of bacterial communities, in which cell communication signals may coordinate the activities of all individual strains. Indeed, a vast array of PGPR populations displaying co-occurring plant-beneficial activities and that may share between each other antagonistic or synergistic effects are interacting with a same host plant (**Figure 3**). When analysing plant growth-promoting effects, it is thus important to integrate the complexity of the interactions between PGPR populations within the rhizo-microbiome. To do so, functional ecology approaches are needed, in which the relations between the size, diversity and activities of PGPR assemblages in the rhizosphere are taken into account. This is of particular importance when assessing the effect of various environmental factors, including that of plant genotype.

### PGPR ECOLOGY IN THE RHIZOSPHERE: FROM INDIVIDUAL STRAINS TO FUNCTIONAL GROUPS

Plant growth-promoting rhizobacteria strains occur in various taxonomic groups, and these different taxonomic groups may be present simultaneously in a given soil (Kyselková et al., 2009; Almario et al., 2013a). This suggests that taxonomically-contrasted PGPR strains may coexist in soil and colonize a same rhizosphere, along with all non-PGPR members of the bacterial community. This possibility has been documented repeatedly, especially when characterizing the taxonomic status of bacterial isolates selected based on their positive effect on plant growth or health, their ability to inhibit phytopathogens, or the occurrence of a particular gene or property of relevance for PGPR effect (Bertrand et al., 2001; Barriuso et al., 2005; Upadhyay et al., 2009). In fact, this possibility seems to be the rule rather than the exception. PGPR populations contributing to a same type of function (i.e., ISR, nitrogen fixation, nutrient solubilization, plant development enhancement, etc.) belong to a same functional group. Functional group approaches can be implemented when specific genes are documented. For instance, nitrogen fixers can be assessed using the *nifH* gene, which encodes the dinitrogenase subunit of the nitrogenase. Its sequence is well conserved within the functional group and it is commonly used as marker to monitor the size and diversity of the diazotrophic community (Poly et al., 2001; Dixon and Kahn, 2004). Some of these PGPR functional groups are taxonomically narrow, such as the *Pseudomonas* DAPG producers (Frapolli et al., 2012). In contrast, others are much more diversified, and certain bacterial functional groups may also comprise both PGPR and non-PGPR strains. For instance, nitrogen fixers include PGPR as well as mutualistic symbionts and even a few pathogens (Herridge et al., 2008).

When considering PGPR-plant relationship in fields, the co-occurrence of genetically contrasted PGPR strains from a same functional group in the rhizosphere has two consequences. First, the activity of a given PGPR functional group corresponds to the resulting contributions of all active individual cells from each type of bacterium within the functional group. If synergistic effects occur between the PGPR populations, the expected performance level for the PGPR function might be higher than if only one type of strain was involved. On this basis, knowing the size of the functional group will help understand the potential importance of the corresponding function. Indeed, for functions leading to enhanced nutrient availability to the plant, such as nitrogen fixation or phosphorus solubilization, the higher the better. For others where optimality matters, such as the production of auxinic signals (Dobbelaere et al., 1999; Spaepen et al., 2007b), the performance level of the functional group will need fine-tuning to avoid production levels too small or too great. How this is ecologically regulated at the scale of the corresponding functional group is unknown, but it raises the possibility of co-evolutionary patterns. To bridge the gap between the potential of a plant-beneficial PGPR function and its actual implementation by PGPR strains, the regulatory effects need also to be taken into consideration. Some of these regulatory effects will be common to all members of the functional group (Prigent-Combaret et al., 2008). However, other regulatory effects may be relevant for a subset only of the functional group. For instance, zinc sulfate stimulates DAPG production in certain but not all genetic groups of *Pseudomonas* PGPR strains (Duffy and Défago, 1999).

Second, the relationships amongst the different PGPR strains co-occurring in a same rhizosphere are important. Interactions will take place within a functional group, as illustrated above with QS regulation of phenazine production in fluorescent *Pseudomonas* PGPR (Pierson et al., 1994). Interactions may also take place between different PGPR functional groups (**Figure 3**), integrating competitive and inhibitory effects (Couillerot et al., 2011), signal jamming (Boyer and Wisniewski-Dyé, 2009) and positive signaling (Combes-Meynet et al., 2011), as well as more indirect processes such as root exudation modifications (Phillips et al., 2004; Dardanelli et al., 2010). These interactions have the potential to modulate spatial colonization patterns of PGPR on roots (Couillerot et al., 2011) and to affect PGPR performance (Pierson et al., 1998). This also suggests that members of different PGPR functional groups can function together, as consortia, with the possibility of synergistic effects or, contrariwise, antagonistic effects. These positive effects may be sought by implementing inoculation procedures in which different types of plant-beneficial microorganisms are used in combination, as highlighted above. Even in this context, interactions between the different microbial strains that are inoculated and indigenous microorganisms (including PGPR) probably matter.

### IMPACT OF PLANT GENOTYPES ON PGPR FUNCTIONAL GROUPS

Plants at species, sub-species and variety levels exhibit substantial genetic and phenotypic diversity (Salamini et al., 2002; Vaughan et al., 2008). In the rhizosphere, different plant genotypes will have a different impact on the number, diversity and activity of microorganisms (Bais et al., 2006; Micallef et al., 2009). This has been shown when comparing different plant species (Grayston et al., 1998; Costa et al., 2006; Berg and Smalla, 2009) or varieties within species (Germida and Siciliano, 2001; van Overbeek and van Elsas, 2008; İnceoǧlu et al., 2010; Bouffaud et al., 2012). It entails differences noticeably in root system structure, root exudation profile, and nutrient acquisition (Czarnota et al., 2003; Comas and Eissenstat, 2009). These effects have also been evidenced when considering microbial functional groups of PGPR or where PGPR predominate.

Nitrogen-fixing bacteria are particularly important for plant nitrogen nutrition (Hsu and Buckley, 2009; Turk et al., 2011). The analysis of functional groups indicated that the size and/or composition of nitrogen-fixing bacteria is influenced by host plant features (**Figure 3**), both at plant species (Perin et al., 2006) and variety levels (Coelho et al., 2009; Wu et al., 2009). Analysis of *nifH* gene transcripts extracted from the rhizosphere showed that only a fraction of the community expresses *nifH*, and that the corresponding bacterial species differed according to the plant variety, pointing to an influence of plant genotype on the functioning of nitrogen-fixing bacteria (Knauth et al., 2005; Mårtensson et al., 2009; Orr et al., 2011). Similar findings were made with the functional group of phosphate solubilizers (Richardson and Simpson, 2011). Their selection by roots varies according to host plant species (Kaeppler et al., 2000; Chen et al., 2002; Ramaekers et al., 2010).

Other functional groups, such as those involved in plant protection from parasites, act mainly by competition or antagonism, even though direct ISR effects might also take place (Weller et al., 2012). For these microorganisms as well, plant genotype can have a major effect on microbial selection processes, as shown with fluorescent pseudomonads producing DAPG (Picard et al., 2004; Bergsma-Vlami et al., 2005; Picard and Bosco, 2006; Frapolli et al., 2010) or hydrogen cyanide (Jamali et al., 2009; Rochat et al., 2010). Plant-genotype differences in rhizosphere ecology may also matter in terms of plant protection efficiency (Smith and Goodman, 1999; Mazzola and Gu, 2002; Mazzola et al., 2004; Ryan et al. 2004).

## CONCLUSION

Plants have evolved different types of biotic interactions with soil microbial populations, ranging from commensalism to mutualism. Within this continuum of interactions, the plant-PGPR cooperation plays a major role by enhancing growth and health of widely diverse plants. Recent progress has helped to understand key features regarding the modes of action and ecology of plant-PGPR interactions, but major knowledge gaps remain. In terms of molecular signaling and functioning, whether PGPR fine-tune plant hormonal pathways similar to those induced by pathogens and symbionts and/or trigger yet-unknown specific pathways requires clarification.

Plant growth-promoting rhizobacteria are able to modulate RSA and *in fine* the vegetative growth and physiology of the whole plant. RSA effects have long been associated with the production of IAA by PGPR. Surprisingly, bacterial modulation of plant auxin distribution and IAA signal transduction pathways, independently of IAA production by PGPR, has also been revealed. It is obviously a step forward in our understanding of plant-PGPR cooperation but it does not fully clarify the bacterial functions and plant hormonal networks involved. Plant hormones regulate genes for the biosynthesis of other hormones or components of hormonal pathways. Consequently, it is possible that PGPR can affect these cross-talks too. It would explain why PGPR can have such pleiotropic effects on plants. One of the major current scientific challenges lying ahead is to understand how these different signaling pathways are integrated to coordinate plant growth and development, and how PGPR influence the plant hormonal network.

Distinct PGPR populations present in a same soil can express plant-beneficial properties in concert. As aforementioned, the relationships between plants and their rhizo-microbiome are complex and vary both according to plant genotypes and soil inhabiting populations (and thus local soil properties, more generally speaking). Next-generation sequencing technologies have started to reveal their taxonomic and functional diversity. They have begun to bring new knowledge on the ecology of PGPR functional groups. In the near future, it is expected that metatranscriptomics and metaproteomics will develop drastically, and will allow further progress on the understanding of the activity and ecological behavior of natural PGPR populations within the rhizosphere. However, given the heterogeneity in space and time of the rhizosphere habitat, samplings at different times and locations within the plant rhizosphere and within fields will be essential to better understand the ecology and performance of PGPR at plant and field plot scales. Nevertheless, despite being very reductionist, mechanistic functional studies using one PGPR and one plant are still useful to investigate the ways PGPR exert beneficial effects on plants. We think that the most important advances on plant-PGPR cooperation will be brought in the future by combining both ecology and functional biology approaches.

## Conflict of Interest Statement

The authors declare that the research was conducted in the absence of any commercial or financial relationships that could be construed as a potential conflict of interest.
